# An analysis of the remission phase in type 1 diabetes within a multiethnic Brazilian sample

**DOI:** 10.1016/j.jped.2024.09.005

**Published:** 2024-11-04

**Authors:** Maria E.N. Ramos, Isabella S. Leão, Joana R.D. Vezzani, Ludmila N.R. Campos, Jorge L. Luescher, Renata S. Berardo, Lenita Zajdenverg, Melanie Rodacki

**Affiliations:** a Universidade Federal do Rio de Janeiro, Faculdade de Medicina, Rio de Janeiro, RJ, Brazil; b Universidade Federal do Rio de Janeiro, Departamento de Medicina Interna, Rio de Janeiro, RJ, Brazil; c Universidade Federal do Rio de Janeiro, Instituto de Puericultura e Pediatria Martagão Gesteira, Rio de Janeiro, RJ, Brazil; d Hospital Federal Servidores do Estado, Departamento de Pediatria, Rio de Janeiro, RJ, Brazil

**Keywords:** Diabetes, Type 1 diabetes, Autoimmunity, Remission phase

## Abstract

**Objective:**

To assess the frequency and potential influencing factors of the remission phase (RP) in Type 1 Diabetes (T1D) as well as the associations between various criteria used for its definition.

**Methods:**

This was a retrospective cohort study based on data collected from medical records. Three criteria were used to evaluate RP: (1) Glycated hemoglobin (HbA1c) < 7.5 % with an insulin dose < 0.5 U/Kg/day, (2) HbA1c < 7 % with an insulin dose < 0.5 U/Kg/day, and (3) Insulin Dose Adjusted A1c (IDAA1c) ≤ 9, calculated as IDAA1c = HbA1c (%) + [4 x insulin dose (U/Kg/day)]. *Statistical analyses included* the Mann-Whitney U Test, Chi-Square Test, and Spearman Correlation.

**Results:**

The sample *consisted of* 144 T1D patients, with a mean age of 26.22 ± 8.30 years and a mean age of onset of 13.30 ± 8.50 years. Of these, 52.9 % were female, 60.3 % were Caucasoid, and 31 % experienced diabetic ketoacidosis (DKA) at disease onset. One year after diagnosis, the occurrence of RP ranged from 27.2 % to 46.8 %, depending on the criteria used. Three patients experienced RP even after seven years, according to criterion 3. No association was found between RP and age, DKA, or pubertal status at onset. The three RP criteria were highly associated with each other (*p* < 0.001).

**Conclusion:**

A significant proportion of patients in this sample experienced RP within 12 months of diagnosis. The three criteria for defining RP were strongly associated, indicating their reliability in both clinical and research contexts.

## Introduction

Type 1 Diabetes (T1D) is caused by immune-mediated destruction of pancreatic beta cells, which produce insulin. After disease onset, some T1D patients enter a period known as the remission phase (RP), or "honeymoon" phase, during which residual beta-cell function is preserved. RP can be complete or partial, with partial RP characterized by satisfactory glycemic control and reduced insulin requirements.^1^ RP has been associated with a better prognosis, including a lower risk of diabetes-related microvascular complications (e.g., retinopathy, nephropathy, neuropathy),^2^ and fewer severe hypoglycemic episodes.^3^

Various definitions of RP have been proposed, which accounts for the variability in RP frequency across studies. The three most commonly used criteria are HbA1c < 7.5 % with insulin dose < 0.5 U/Kg/day; the International Society for Pediatric and Adolescent Diabetes (ISPAD) criteria of HbA1c < 7 % with insulin dose < 0.5 U/Kg/day (4); and the Insulin Dose Adjusted A1c (IDAA1c) index, which is the most widely accepted definition due to its strong correlation with C-peptide levels, a marker of insulin secretion.^1^

Several factors may influence the duration of RP, including age of onset, DKA presence, islet autoantibodies, and gender.^1^^,^^2^^,^^4^, ^5^, ^6^, ^7^, ^8^, ^9^ Younger age at diagnosis and DKA onset have been associated with a lower likelihood of entering RP.^4^^,^^9^ Some studies suggest males are more likely to experience RP.^6^^,^^7^^,^^9^, though others found no significant gender differences.^5^

The pubertal stage at onset could also be an influencing factor for the development of RP in T1D. Patients with a prepubertal disease onset present a more aggressive course of the disease, while those with pubertal patients usually present a milder autoimmune response, which is probably related to the age of the disease onset. However, glycemic control tends to worsen during puberty, especially in females. This is probably explained by the influence of sexual hormones on insulin sensitivity, as well as other behavioral factors.^10^ Body mass index (BMI) and RP may also have a relationship. According to the "acceleration theory," higher BMI increases insulin resistance, leading to earlier diagnosis and insulin therapy, which might favor RP development.^11^

Most studies have focused on Caucasian populations, and it is important to investigate whether these factors also affect RP frequency and duration in multiethnic populations. Brazil, known for its ethnic diversity, presents an opportunity to explore these dynamics. Recent therapeutic advances with disease-modifying agents make understanding RP particularly relevant. The anti-CD3 monoclonal antibody Teplizumab has been associated with extended RP and improved prognosis in preclinical T1D.^12^ Verapamil, traditionally used for hypertension, has shown promise in increasing C-peptide secretion and prolonging RP.^13^ This study aimed to examine the frequency of RP, the factors influencing it, and the association between different criteria in a multiethnic Brazilian population.

## Subjects and methods

### Study design

This was a retrospective study based on epidemiological and clinical data collected from January 2020 to March 2022. The present sample consisted of Brazilian T1D outpatients who were being followed up at a Diabetes Outpatient Clinic from a University hospital. Data were obtained from patient medical records and telephone contact.

### Ethical aspects

All patients participating in this study signed the Informed Consent Form and the project was approved by the institutional ethical committee at the protocol number 37,001,514.0.0000.5257 and 088,766/2014.

### Selection criteria

Patients with a confirmed diagnosis of T1D, according to the American Diabetes Association (ADA) criteria, who were followed at the hospital's Diabetes Outpatient Clinic were included. All patients had at least one measurement of Glycated Hemoglobin (HbA1c) and data on insulin dosage at disease onset, as well as at 6, 12, 18, 24, 30, and 36 months, and 4, 5, 6, 7, 8, 9, and 10 years after onset. Patients were excluded if HbA1c or insulin dose data were missing due to the absence of laboratory tests or medical consultations during follow-up.

### Research procedures

The authors collected data on age, gender, ethnicity, age at onset, and the presence of DKA at T1D onset from medical records. For ethnicity determination, data recorded by the attending physician were used, and the population was categorized into Caucasoid and Non-Caucasoid groups, with the latter primarily consisting of Afro-Brazilians. Information on HbA1c and insulin dose (U/Kg/day) was also collected at disease onset and at specified intervals. HbA1c levels were measured according to the National Glycohemoglobin Standardization Program (NGSP).

### Remission phase definition

In this study, the authors assessed the frequency of partial remission phase (RP) based on three criteria: (1) HbA1c < 7.5 % with insulin dose < 0.5 U/Kg/day, referred to as remission 1 (R1);^14^ (2) HbA1c < 7 % with insulin dose < 0.5 U/Kg/day, referred to as remission 2 (R2), as defined by the International Society for Pediatric and Adolescent Diabetes (ISPAD);^15^ and (3) Insulin Dose Adjusted A1c (IDAA1c) ≤ 9, calculated using the formula: IDAA1c = HbA1c (%) + [4 × insulin dose (U/Kg/day)], referred to as remission 3 (R3), as proposed by Mortensen et al.^16^

### Puberty definition

Pubertal status at disease onset was evaluated using the Tanner scale of sexual maturity. Breast development and pubic hair growth were considered for girls, while pubic hair and genital development were considered for boys.^10^

### Statistical analysis

Statistical analyses were performed using the SPSS software, with a significance level set at *p* < 0.05. Mann-Whitney U and Chi-Square tests were used to compare continuous and categorical variables between groups, respectively. Descriptive statistical analysis was performed, and results are presented as means and standard deviation (SD) The data had a non-normal distribution.

## Results

### Study population

Data were collected from 321 outpatients, of which 144 patients were selected for the study, while 177 were excluded due to the absence of HbA1c or insulin dose records in their medical files. The mean age was 26.22 ± 8.30 years, and the mean age of onset was 13.30 ± 8.50 years (Table 1). Most of the participants were female (*N* = 74, 52.9 %) and Caucasoid (*N* = 82, 60.3 %). At disease onset, 87 patients presented with polyphagia, polydipsia, and polyuria (*N* = 87, 60.4 %), while 40 patients experienced DKA (*N* = 40, 31.0 %). Only 2 patients presented with neither condition (*N* = 2, 1.4 %) (Table 1).

**Table 1:** Sample characteristics: continuous and categorical variables.

Variable	N (%)
Gender	
Female	74 (52.9)
Male	66 (47.1)
Total	140 (100)
Ethnicity	
Caucasoids	82 (60.3)
Non-Caucasoids	54 (39.7)
Total	136 (100)
DKA at onset	
Yes	40 (31.0)
No	89 (69.0)
Total	129 (100)
Age	M (SD)
Current age (y/o)	26.22 (8.30)
Age of onset (y/o)	13.30 (8.50)

% valid percentage of cases, DKA, Diabetic ketoacidosis; M, mean; N, frequency of cases; SD, standard deviation; Y/O, years old,.

### RP frequency and association between criteria

The frequency of partial RP, according to criteria 1, 2, and 3, at 12, 24 months, 5, and 7 years is presented in Table 2. A strong association was found between all criteria (*p* < 0.001), as well as between criteria 1 or 2 and criterion 3 at both 12 and 24 months (*p* < 0.001 for all associations).

**Table 2:** Frequency of remission phase according to criteria (RP1), criteria 2 (RP2) and criteria 3 (RP3) after 12 and 24 months, 5 and 7 years.

	After 12 months	After 24 months	After 5 years	After 7 years
	N (%)	N (%)	N (%)	N (%)
RP1				
Presence	27 (33.3)	8 (10.1)	1 (1.5)	–
Absence	54 (66.7)	71 (89.9)	64 (98.5)	63 (100)
Total	81 (100)	79 (100)	65 (100)	63 (100)
RP2				
Presence	22 (27.2)	5 (6.3)	–	–
Absence	59 (72.8)	74 (93.7)	66 (100)	62 (100)
Total	81 (100)	79 (100)	66 (100)	62 (100)
RP3				
Presence	37 (46.8)	17 (21.8)	2 (3.1)	3 (4.8)
Absence	42 (53.2)	61 (78.2)	63 (96.9)	60 (95.2)
Total	79 (100)	78 (100)	65 (100)	63 (100)

N, frequency of cases; RP, Remission phase.

### Factors influencing the remission phase

In this study, the authors examined the influence of four factors on the frequency and duration of RP: age of onset, presence of DKA at onset, ethnicity, and puberty. Mann-Whitney test was used to compare the age at onset between those who developed RP and others. For the remaining three categorical variables, Chi-Square tests were used. Patients diagnosed before puberty had a lower frequency of RP defined by criterion 1 compared to others (0 vs 7; *p* = 0.038) at 24 months. Regarding ethnicity, Caucasoid patients had a higher likelihood of developing RP according to criterion 2 compared to others (*p* = 0.039 at 24 months). Glycemic control, as measured by HbA1c levels, was similar between Caucasoid and non-Caucasoid groups at 12, 24 months, and 5 and 7 years (*p* = 0.399, *p* = 0.420, *p* = 0.155, and *p* = 0.448, respectively). No other associations were found between potential influencing factors, such as age of onset or presence of DKA at onset, and the development of RP.

## Discussion

This retrospective study evaluated the frequency of RP in patients with T1D within the Brazilian population and explored factors influencing its development and duration. It also examined the associations between different criteria used to define RP.

Firstly, partial RP was observed in a significant proportion of patients one year after T1D onset, placing this population at an intermediate position compared to other countries. There is considerable variation in RP frequency across different diabetes centers. In a multicenter study by Pozzilli et al.^17^, involving 12 European centers across various countries, a sample of 189 patients aged 5 to 35 years was followed for 36 months. This study used a consistent criterion to define RP (no insulin use with normal HbA1c measured at three separate intervals, each three months apart), allowing for reliable comparison. RP frequency varied widely, from 12.2 % in Germany to 90 % in Poland, though the authors could not identify clear reasons for these differences. Interestingly, in the present study, partial RP was observed even several years after T1D onset (2 and 3 cases after 5 and 7 years, respectively, using criterion 3). Studies investigating such long-term RP progression are rare, making these findings of particular interest. None of the patients in this study experienced complete RP.

All criteria used for RP definition in this study were highly correlated, indicating their applicability in both clinical and research settings. This information could be useful for future intervention studies aimed at curing diabetes or preserving beta-cell function. It would also be valuable to assess the association with other commonly used RP criteria, such as HbA1c ≤ 6.5 % + insulin dose ≤ 0.4 U/kg^18^ or C-peptide ≥ 300 pmol/L.^1^ Although the criteria based on insulin dose and HbA1c are simpler, cheaper, and more readily available, C-peptide measurement is a reliable method for assessing residual beta-cell function.^1^

The authors found no significant association between age at T1D onset and RP during the follow-up period. Some studies suggest that individuals diagnosed at a younger age are less likely to develop RP,^4^^,^^9^^,^^19^^,^^20^ while others have found no such association.^5^^,^^7^^,^^21^ Moreover, there was no association between pubertal status at T1D onset and RP development, except for partial RP at 24 months, where patients diagnosed before puberty had a lower RP frequency according to criterion 1. Dost et al.^4^ and Böber et al.^21^ have shown that children with more advanced Tanner stages are more likely to experience RP, likely due to a stronger autoimmune response against beta cells in younger patients. Conversely, Kara et al.^6^ found a higher frequency of RP in prepubertal children, which may be explained by poorer glycemic control during adolescence and reduced insulin sensitivity caused by hormonal changes.

The relationship between DKA and RP was also analyzed. While previous studies^5^^,^^9^^,^^21^^,^^22^ have shown that patients with DKA at onset are less likely to experience RP, the authors did not observe this association. The divergent results in this study could be attributed to the older mean age of diagnosis in the sample compared to studies that focused on younger populations. The present sample consisted mainly of adult patients, with a mean age of 26.22 years and a mean age of onset of 13.3 years, while most other studies included pediatric populations, which may have influenced disease progression and glycemic control, typically worse during adolescence. Ethnic differences could also explain these discrepancies.

The authors observed no difference in glycemic control between Caucasoid and non-Caucasoid patients during follow-up. Interestingly, Caucasoid patients were more likely to develop RP compared to non-Caucasoids. Previous studies have suggested the influence of ethnicity on RP behavior.^19^^,^^21^ This finding is noteworthy, as few studies have specifically examined the role of ethnicity in RP.^19^^,^^21^ However, it is crucial to determine whether these results may be confounded by socioeconomic factors.

This study has several limitations. First, it is reasonable to critique the term "remission phase" as applied to the definitions tested. While patients meeting these criteria do achieve good glycemic control with reduced insulin requirements, likely reflecting transient beta-cell functional recovery, no histopathological confirmation was performed. Additionally, one of the RP definitions (criterion 1) used HbA1c < 7.5 % as an indicator of adequate control, despite this value being above the generally recommended target for diabetes management. Another limitation was the missing data in medical records, which reduced the sample size and long-term data availability, particularly at 5 and 7 years. Potential confounding factors, such as BMI and socioeconomic status, were not assessed. Moreover, C-peptide, a more direct measure of beta-cell function, was not routinely analyzed in the studied center, although the correlation between C-peptide and IDAA1c is well established.

Nevertheless, the present study provides important insights into the field. It contributes data on the epidemiological behavior of RP and its influencing factors within the multiethnic Brazilian population and offers longer-term follow-up information than is typically available (5 and 7 years of T1D duration). Additionally, the authors demonstrated a strong association between the three RP criteria analyzed, offering a solid basis for safer comparisons between studies in this field.

In conclusion, the authors observed RP in a significant proportion of patients up to 24 months after T1D onset, with a strong association between the three criteria used to define RP. Caucasoid patients were more likely to develop RP, though it remains unclear whether this reflects ethnic differences in T1D behavior or socioeconomic disparities. In this sample, neither DKA nor age at onset were related to RP development, but patients diagnosed after the onset of puberty were less likely to develop partial RP at 24 months. Larger, long-term prospective studies in multiethnic populations are needed to confirm these findings and to further explore the role of ethnicity in the evolution of RP and residual beta-cell function.

## Author contributions

Isabella Sued Leão and Maria Eduarda Nascimento researched the data, and Maria Eduarda Nascimento analyzed the data and wrote the manuscript. Joana Rodrigues and Ludmilla Nascimento selected the patients and researched the data. Melanie Rodacki, Lenita Zajdenverg, Jorge Luescher and Renata Berardo reviewed and edited the manuscript.

## Conflicts of interest

The authors declare no conflicts of interest.
